# Editorial: Advances in the use of neuromonitoring in newborns

**DOI:** 10.3389/fped.2023.1215991

**Published:** 2023-05-22

**Authors:** Valerie Y. Chock, Krisa P. Van Meurs

**Affiliations:** Division of Neonatal and Developmental Medicine, Stanford University School of Medicine, Palo Alto, CA, United States

**Keywords:** near-infrared spectroscopy (NIRS), amplitude integrated electroencephalography (aEEG), continuous electroencephalography (CEEG), neonate, brain

**Editorial on the Research Topic** Advances in the use of neuromonitoring in newborns

## Introduction

**Figure 1:**
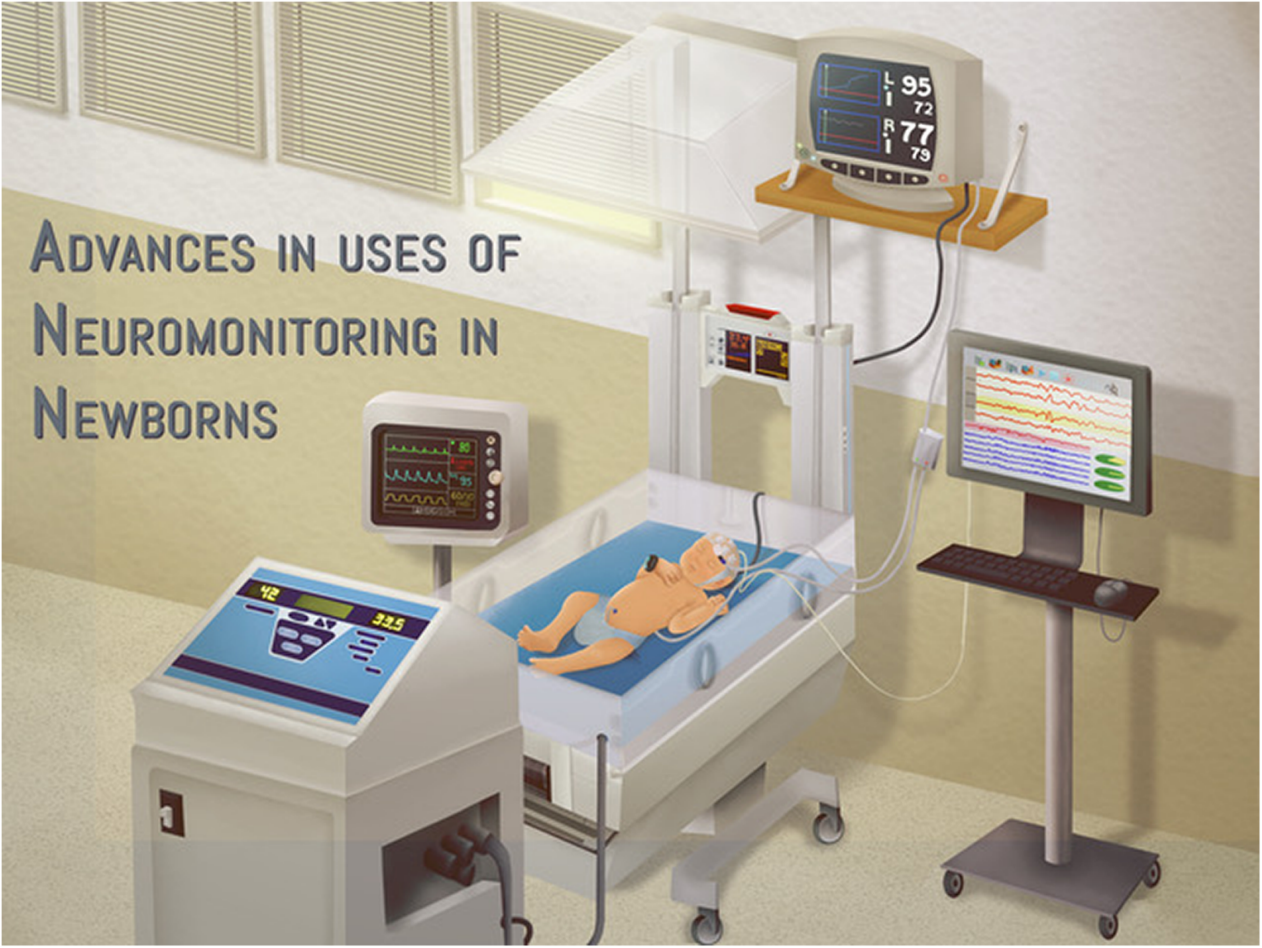
Advances in bedside neuromonitoring techniques continue to enhance neonatal neurocritical care.

Neonatal neurocritical care is a rapidly evolving subspeciality with the goal of implementing neuroprotective strategies and identifying new therapies to care for babies at risk of or with existing brain injury in order to improve long term neurodevelopmental outcomes. Brain-focused care is a desired development following decades of focus on survival and extending the limits of viability. Neonatal neurocritical care and Neuro-NICUs seek to better address the sizable population at risk of or with brain injury by integrating intensive care practices with focused neurologic care. Bedside continuous neuromonitoring has a significant role in this ambitious endeavor with specific focus on near-infrared spectroscopy (NIRS), continuous electroencephalography (cEEG), and amplitude integrated electroencephalography (aEEG) as well as the combined use of these and other techniques called multimodal neuromonitoring (Figure 1). This collection of articles focuses on the exciting developments in the field of neonatal neuromonitoring.

### The present status and future of neonatal neuromonitoring

Variane et al. comprehensively describe the current uses of neuromonitoring in Neuro-NICUs focusing on aEEG, cEEG, and NIRS. Multimodality monitoring simultaneously with aEEG or cEEG and NIRS is likely to improve understanding of the physiology of both functional and hemodynamic changes and the resulting risk of cerebral injury. The incorporation of other time-synchronized physiologic vital signs will enhance the development of neuroprotective strategies. The available options, as well as challenges of data integration and processing, are described and the power of artificial intelligence and machine learning to significantly benefit vulnerable critically ill neonates is highlighted. The authors conclude that the broader use of brain monitoring with analysis of large amounts of clinical data available in an intensive care setting has the potential to significantly change care and improve outcomes.

### Hypoxic ischemic encephalopathy (HIE)

Given the significant risk for adverse outcomes in newborns with HIE, including death and neurodevelopmental impairment, a wide range of neurodiagnostic modalities have been evaluated to provide critical diagnostic and prognostic information on brain injury and prediction of outcome. Chock et al. provide a comprehensive review of continuous measures including cEEG, aEEG, NIRS, and heart rate variability as well as serial measures including cranial ultrasound and somatic and visual evoked potentials in newborns with HIE undergoing therapeutic hypothermia. cEEG and aEEG are promising predictors of adverse outcome with high specificity and sensitivity. The higher specificity seen with cEEG is offset by its greater complexity and cost for placement and interpretation. Mutli-modality use of both cEEG or aEEG with NIRS along with physiologic vital signs is appealing as it allows for continuous bedside assessment of both cerebral function and cerebral oxygenation and correlates them with alterations in other physiologic parameters.

### Prematurity

NIRS monitoring has been investigated in premature infants in different situations including the transitional period after birth (1–4), for diagnosis of a hemodynamically significant patent ductus arteriosus (5), or for intraventricular hemorrhage or post-hemorrhagic hydrocephalus (6, 7). Several observational studies have also demonstrated an association between early cerebral hypoxia and adverse outcomes including neurodevelopment (4, 8–10). While the SafeBoosC randomized clinical trial (RCT) demonstrated a reduction in the burden of cerebral hypoxia using a NIRS interventional guideline to maintain targeted cerebral saturation values (1), current evidence from large, multinational RCTs does not demonstrate a reduction in adverse outcomes including mortality or brain injury (2, 3, 11). Greisen et al. provide a perspective on the state of NIRS monitoring for the preterm infant. The authors compare cerebral oximetry to other monitoring modalities that may be used in the neonatal intensive care setting despite minimal empiric evidence of clinical benefit, including pulse oximetry, non-invasive electric cardiometry, and invasive blood pressure monitoring. Future research may require a focus on more granular effects of a targeted cerebral NIRS algorithm on the brain and clarify timing and conditions for which NIRS monitoring may best optimize care for the premature infant.

### Congenital heart disease (CHD)

Neuromonitoring with NIRS may further have utility in infants with CHD, particularly in the peri-operative period and may reduce the need for intubations in select infants with CHD (12, 13). Cerebral hypoxia has been associated with adverse neuroimaging outcomes and impaired neurodevelopment in the CHD population (14–16). Hoffman et al. describe the utility of both cerebral and somatic NIRS monitoring with relation to carbon dioxide tension in 178 infants with hypoplastic left heart syndrome following Norwood palliation. Increasing arterial carbon dioxide tension was associated with increased cerebral but decreased renal oxygenation. Along with this differential response to carbon dioxide, the magnitude of these responses was greater in the early 18 h post-operative period. Given the sensitivity of the brain to hypocarbia with resulting cerebral vasoconstriction, strict attention to carbon dioxide levels may be an important strategy to optimize cerebral blood flow. The authors speculate that normalization of cerebral hemodynamics may be achieved in the early post-operative period with dynamic manipulation of carbon dioxide levels through directed ventilator approaches such as permissive hypercapnia.

### Newer indications for multimodal monitoring

Continuous neuromonitoring using NIRS and aEEG or cEEG allows for bedside assessment of brain, renal, and splanchnic/mesenteric oxygenation and perfusion as well as cerebral function and seizure detection. Further combination with vital sign monitoring including blood pressure, pulse oximetry, heart rate and temperature is described as multimodal monitoring and provides further understanding of physiology. These approaches have been previously described in premature infants and in newborns with HIE (4, 17, 18). Variane et al. describe 10 additional cases including cardiopulmonary disorders, circulatory disorders, and abdominal disorders, where multimodal monitoring with multi-site NIRS and in some cases with aEEG provided earlier recognition of the underlying hemodynamic status and its impact.

### Cerebral autoregulation

Impairment of cerebral autoregulation may lead to alterations in cerebral blood flow, particularly in vulnerable populations including neonates with HIE, preterm infants, and those with congenital heart disease. Leon et al. review various modalities to assess cerebral blood flow and specifically cerebral autoregulatory capacity in these conditions. Doppler ultrasound, magnetic resonance imaging, and NIRS are techniques that have advantages and limitations for use in the fetal and neonatal period. The authors emphasize that future assessment of cerebral autoregulation will require an individualized approach and use of multiple synchronized monitoring modalities and predictive analytics.

### Current and future uses of continuous EEG

Several uses for cEEG in the neonatal population have been described, including seizure diagnosis and management, identifying neonates at risk for seizures, and prognostication by assessment of background activity. It is the standard of care for seizure diagnosis as designated by the World Health Organization (WHO), International League Against Epilepsy (ILAE), and American Clinical Neurophysiology Society (ACNS) (19–21). Sandoval et al. acknowledge that high cost is a factor limiting more widespread use of cEEG. In comparison, aEEG is recognized as often helpful when used together with cEEG, as it allows bedside providers to review the simplified tracing without the need for neurophysiologists. However, short, low amplitude seizures or seizures in areas of the brain not monitored by aEEG will be missed, and a high skill level with aEEG interpretation is required. Future uses of cEEG include centralized cEEG interpretation, automated seizure detection, and prenatal EEG use.

## Conclusion

This collection of original articles and reviews on neonatal neuromonitoring adds to our current understanding of the techniques available to non-invasively assess the newborn brain. While EEG and NIRS have been mainstays of neuromonitoring, newer indications for these techniques including applications in different neonatal populations have been described. Research studies have also refined the optimal timing of monitoring and highlight an outcomes-based focus. The approach to combined or multimodal neuromonitoring with time-synchronized vital signs has been evolving with the potential for remote-monitoring and automated detection of concerning events such as seizures or cerebral hypoxia. Future advances may incorporate additional physiologic parameters such as heart rate variability or point-of-care imaging and utilize artificial intelligence and machine learning to interrogate multiple bedside data streams. Progress in the field of neuromonitoring will continue to improve predictive capabilities and guide clinical management with the goal of optimizing brain health and development in the newborn.

## References

[1] Hyttel-Sorensen S Pellicer A Alderliesten T Austin T van Bel F Benders M Cerebral near infrared spectroscopy oximetry in extremely preterm infants: phase II randomised clinical trial. Br Med J. (2015) 350:g7635. 10.1136/bmj.g7635 25569128 PMC4283997

[2] Hansen ML Pellicer A Hyttel-Sørensen S Ergenekon E Szczapa T Hagmann C Cerebral oximetry monitoring in extremely preterm infants. N Engl J Med. (2023) 388:1501–11. 10.1056/NEJMoa2207554 37075142

[3] Pichler G Goeral K Hammerl M Perme T Dempsey EM Springer L Cerebral regional tissue oxygen saturation to guide oxygen delivery in preterm neonates during immediate transition after birth (COSGOD III): multicentre randomised phase 3 clinical trial. Br Med J. (2023) 380:e072313. 10.1136/bmj-2022-072313 36693654 PMC9871806

[4] Katheria AC Stout J Morales AL Poeltler D Rich WD Steen J Association between early cerebral oxygenation and neurodevelopmental impairment or death in premature infants. J Perinatol. (2021) 41:743–8. 10.1038/s41372-021-00942-w 33589727 PMC7883949

[5] Chock VY Rose LA Mante JV Punn R. Near-infrared spectroscopy for detection of a significant patent ductus arteriosus. Pediatr Res. (2016) 80:675–80. 10.1038/pr.2016.148 27603562

[6] Alderliesten T Lemmers PMA Smarius JJM van de Vosse RE Baerts W van Bel F. Cerebral oxygenation, extraction, and autoregulation in very preterm infants who develop peri-intraventricular hemorrhage. J Pediatr. (2013) 162:698–704.e2. 10.1016/j.jpeds.2012.09.038 23140883

[7] June A Heck T Shah TA Vazifedan T Bass WT. Decreased cerebral oxygenation in premature infants with progressive posthemorrhagic ventricular dilatation may help with timing of intervention. Am J Perinatol. (2021). 10.1055/s-0041-1736533. [Epub ahead of print] 34674212

[8] Chock VY Kwon SH Ambalavanan N Batton B Nelin LD Chalak LF Cerebral oxygenation and autoregulation in preterm infants (early NIRS study). J Pediatr. (2020) 227:94–100.e1. 10.1016/j.jpeds.2020.08.036 32818482

[9] El-Dib M Munster C Sunwoo J Cherkerzian S Lee S Hildrey E Association of early cerebral oxygen saturation and brain injury in extremely preterm infants. J Perinatol. (2022) 42:1385–91. 10.1038/s41372-022-01447-w 35790852 PMC11262415

[10] Alderliesten T van Bel F van der Aa NE Steendijk P van Haastert IC de Vries LS Low cerebral oxygenation in preterm infants is associated with adverse neurodevelopmental outcome. J Pediatr. (2019) 207:109–116.e2. 10.1016/j.jpeds.2018.11.038 30577979

[11] Plomgaard AM Schwarz CE Claris O Dempsey EM Fumagalli M Hyttel-Sorensen S Early cerebral hypoxia in extremely preterm infants and neurodevelopmental impairment at 2 year of age: a post hoc analysis of the SafeBoosC II trial. PLoS One. (2022) 17:e0262640. 10.1371/journal.pone.0262640 35073354 PMC8786171

[12] Johnson BA Hoffman GM Tweddell JS Cava JR Basir M Mitchell ME Near-infrared spectroscopy in neonates before palliation of hypoplastic left heart syndrome. Ann Thorac Surg. (2009) 87:571–7; discussion 577–79. 10.1016/j.athoracsur.2008.10.043 19161781

[13] Cheng HH Ferradal SL Vyas R Wigmore D McDavitt E Soul JS Abnormalities in cerebral hemodynamics and changes with surgical intervention in neonates with congenital heart disease. J Thorac Cardiovasc Surg. (2020) 159:2012–21. 10.1016/j.jtcvs.2019.08.045 31685276 PMC7699439

[14] Dent CL Spaeth JP Jones BV Schwartz SM Glauser TA Hallinan B Brain magnetic resonance imaging abnormalities after the norwood procedure using regional cerebral perfusion. J Thorac Cardiovasc Surg. (2005) 130:1523–30. 10.1016/j.jtcvs.2005.07.051 16307993

[15] Hoffman GM Brosig CL Mussatto KA Tweddell JS Ghanayem NS. Perioperative cerebral oxygen saturation in neonates with hypoplastic left heart syndrome and childhood neurodevelopmental outcome. J Thorac Cardiovasc Surg. (2013) 146:1153–64. 10.1016/j.jtcvs.2012.12.060 23317941

[16] Sood ED Benzaquen JS Davies RR Woodford E Pizarro C. Predictive value of perioperative near-infrared spectroscopy for neurodevelopmental outcomes after cardiac surgery in infancy. J Thorac Cardiovasc Surg. (2013) 145:438–445.e1; discussion 444–45. 10.1016/j.jtcvs.2012.10.033 23219333

[17] Ancora G Maranella E Grandi S Sbravati F Coccolini E Savini S Early predictors of short term neurodevelopmental outcome in asphyxiated cooled infants. A combined brain amplitude integrated electroencephalography and near infrared spectroscopy study. Brain Dev. (2013) 35:26–31. 10.1016/j.braindev.2011.09.008 22082686

[18] Goeral K Urlesberger B Giordano V Kasprian G Wagner M Schmidt L Prediction of outcome in neonates with hypoxic-ischemic encephalopathy II: role of amplitude-integrated electroencephalography and cerebral oxygen saturation measured by near-infrared spectroscopy. Neonatology. (2017) 112:193–202. 10.1159/000468976 28704822

[19] Shellhaas RA Chang T Tsuchida T Scher MS Riviello JJ Abend NS The American clinical neurophysiology society’s guideline on continuous electroencephalography monitoring in neonates. J Clin Neurophysiol. (2011) 28:611–7. 10.1097/WNP.0b013e31823e96d7 22146359

[20] World Health Organization. Guidelines on neonatal seizures. Geneva: World Health Organization (2011).

[21] Pressler RM Cilio MR Mizrahi EM Moshé SL Nunes ML Plouin P The ILAE classification of seizures and the epilepsies: modification for seizures in the neonate. Position paper by the ILAE task force on neonatal seizures. Epilepsia. (2021) 62:615–28. 10.1111/epi.16815 33522601

